# Assessment of hyperspectral imaging and CycleGAN-simulated narrowband techniques to detect early esophageal cancer

**DOI:** 10.1038/s41598-023-47833-y

**Published:** 2023-11-22

**Authors:** Kai-Yao Yang, Arvind Mukundan, Yu-Ming Tsao, Xian-Hong Shi, Chien-Wei Huang, Hsiang-Chen Wang

**Affiliations:** 1 Department of Gastroenterology, Kaohsiung Armed Forces General Hospital, 2, Zhongzheng 1st Rd., Lingya District, Kaohsiung, 80284 Taiwan; 2 Department of Mechanical Engineering, National Chung Cheng University, 168, University Rd., Min Hsiung, 62102 Chiayi Taiwan; 3 Department of Nursing, Tajen University, 20, Weixin Rd., Yanpu, 90741 Pingtung Taiwan; 4 Hitspectra Intelligent Technology Co., Ltd., 4F., No. 2, Fuxing 4th Rd., Qianzhen District, Kaohsiung, 80661 Taiwan; 5 Department of Medical Research, Dalin Tzu Chi General Hospital, 2, Min-Sheng Rd., Dalin, 62247 Chiayi Taiwan

**Keywords:** Biotechnology, Cancer, Biomarkers, Gastroenterology, Optics and photonics

## Abstract

The clinical signs and symptoms of esophageal cancer (EC) are often not discernible until the intermediate or advanced phases. The detection of EC in advanced stages significantly decreases the survival rate to below 20%. This study conducts a comparative analysis of the efficacy of several imaging techniques, including white light image (WLI), narrowband imaging (NBI), cycle-consistent adversarial network simulated narrowband image (CNBI), and hyperspectral imaging simulated narrowband image (HNBI), in the early detection of esophageal cancer (EC). In conjunction with Kaohsiung Armed Forces General Hospital, a dataset consisting of 1000 EC pictures was used, including 500 images captured using WLI and 500 images captured using NBI. The CycleGAN model was used to generate the CNBI dataset. Additionally, a novel method for HSI imaging was created with the objective of generating HNBI pictures. The evaluation of the efficacy of these four picture types in early detection of EC was conducted using three indicators: CIEDE2000, entropy, and the structural similarity index measure (SSIM). Results of the CIEDE2000, entropy, and SSIM analyses suggest that using CycleGAN to generate CNBI images and HSI model for creating HNBI images is superior in detecting early esophageal cancer compared to the use of conventional WLI and NBI techniques.

## Introduction

Esophageal cancer (EC) is one of the deadliest cancers which is predominant in the countries in Africa, North America and south east Asia. Currently, it is the sixth most deadly cancer with the 5-year survival rate as low as 15%^1,2^ while it is the seventh most commonly found cancer in the world with more than 500,000 deaths every year^3,4^. EC can be classified into two common types; namely esophageal squamous cell carcinoma (ESCC) and esophageal adenocarcinoma (EAC)^5^. The most common type of EC is ESCC which accounts to approximately 90% cases over the world^6,7^. Although, recent trends suggests that EAC has been increasing steadily^8^.

With the increase in the cancer occurrences and the poor survival rate, detection and immediate personalized treatment of EC during its earlier stage is very important^9^. Even with many developments in cancer detection techniques if EC is not detected in its early stages, the survival rate of the patients drops to less than 20%^10^. However, if the cancer has been detected at an earlier stage the survival rate can be improved drastically^11,12^. The endoscopists are unable to spot the EC in during its initial phase because there are no specific symptoms which suggests the presence of EC^13,14^. Therefore, the use of artificial engineering (AI) or biomarkers based on bio-sensors are required for detecting EC in the earlier stages^15–18^.

Ghatwary et al., developed a new deep learning (DL) model based on faster region-based convolutional neural network (Faster R-CNN) and gabor handcrafted features automatically to detect esophageal anomalies from endoscopic pictures^19^. Yang et al., trained a deep convolutional neural network (DCNN) using 798 PET images for early detection of ESCC^20^. In their research, Wang et al. used a deep convolutional neural network (CNN) combined with a single-shot multibox detector (SSD) to effectively identify early EC^21^. Li et al. conducted a study to examine the viability of using serum-enhanced Raman spectroscopy in combination with silver nanoparticles (Ag NPs) and a support vector machine (SVM) for the purpose of distinguishing between individuals with EC and those without the disease^22^. The investigation of semantic segmentation has been conducted to explore its potential for early identification and categorization of esophageal cancer^23^. Although CAD, RGB image processing, and biosensors have been used for cancer diagnosis and have shown their effectiveness in giving valuable information, it is important to acknowledge that these systems also have some drawbacks. In the realm of CAD, the conventional approach necessitates the use of substantial quantities of aggregated training data and computing resources in order to achieve enhanced machine learning performance^24^. This is mostly due to the fact that traditional CAD systems only employ three color channels, namely red, green, and blue. The effectiveness of CAD models in cancer detection is hindered by a technical constraint identified in a recent CAD model for colonoscopy, which used a limited dataset^25^. One potential approach to address the many issues is the integration of HSI with CAD approaches. The possible enhancement of cancer detection performance may be achieved by using a broad range of light, spanning from ultraviolet (UV) to far-infrared, for picture capture, rather than relying on the usual RGB color model. The use of a noninvasive methodology yields a more extensive range of information pertaining to the topic matter, as shown by empirical study outcomes. However, only with white light imaging (WLI) alone it is impossible to diagnose the EC at the early stages. However, HSI combined with narrow-band imaging (NBI) is a technique used to enhance the image for early EC detection^26,27^. HSI has many applications including air pollution detection^28,29^, cancer detection^30–33^, counterfeit detection^34–36^, aerospace engineering^37–40^, military^41,42^, agriculture^43–45^, remote sensing^46,47^, quality detection^48,49^, microbiology^50,51^ among others.

Therefore, this study compares the performance of WLI, NBI, CycleGAN CNBI, HNBI to detect esophageal cancer in its early stages. The results of the CIEDE2000, entropy and SSIM analysis suggests that the use of CycleGAN to generate CNBI and HNBI images were superior detect esophageal cancer when compared with normal WLI and NBI. The key contribution and findings from this study are:

- Advanced imaging techniques: introduction and evaluation of cutting-edge imaging techniques, including HSI and CNBI, for early EC detection.
- Comparative analysis: comprehensive assessment of the effectiveness of four distinct image types (WLI, NBI, CNBI, and HNBI) in the context of early EC detection, shedding light on their relative performance.
- Practical applications: exploration of the potential applications of CycleGAN-simulated NBI images in AI-driven object detection, streamlining image collection, and preprocessing while reducing patient discomfort and procedure time, especially in the context of newer endoscopic cameras like capsule endoscopes.
- Improved medical practices: the potential to enhance doctors' recognition rates, providing patients with a higher level of comfort, and enabling earlier symptom detection through the continued development and implementation of advanced imaging technology.
- Interdisciplinary insights: bridging the gap between advanced imaging techniques and medical practice, offering a valuable interdisciplinary perspective with far-reaching implications for early disease detection and patient care.

## Materials and methods

**Figure 1:**
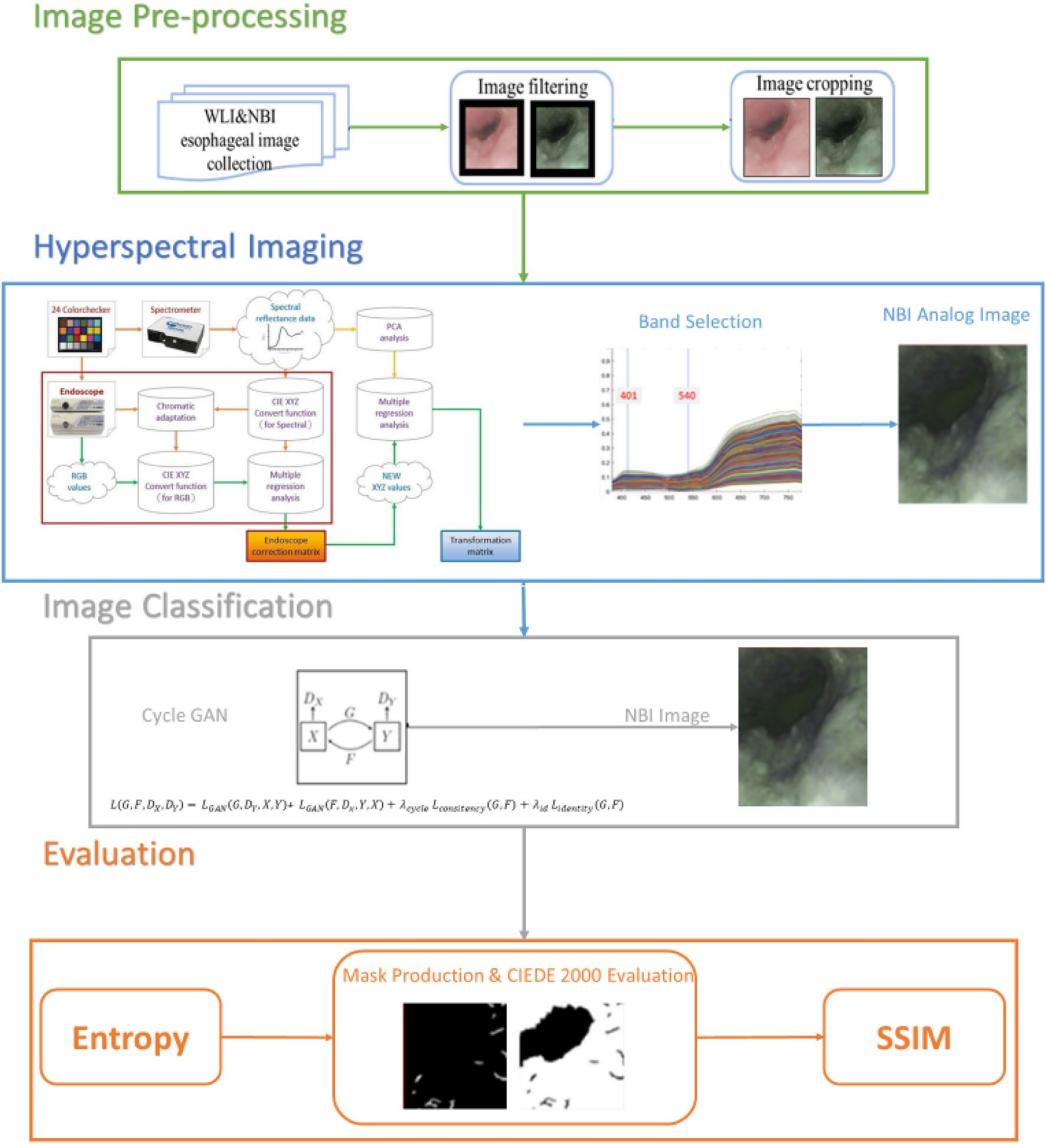
Flowchart of research architecture. It includes data acquisition, preprocessing, spectrum conversion, hyperspectral image simulation narrow-band, CycleGAN narrow-band simulation, objective index evaluation.

In this study, we conducted an analysis of esophageal cancer utilizing a dataset consisting of 1000 images. This dataset included 500 images captured using WLI and another 500 images captured using NBI. These images were sourced from the Kaohsiung Armed Forces General Hospital. While this number may appear limited in certain contexts, it is important to note that the dataset was carefully curated, focusing on high-quality images and relevant criteria to ensure its suitability for our study. The sample size was determined based on the availability of high-quality medical images meeting our research objectives. Although larger datasets are valuable, the quality and relevance of the data often take precedence in medical imaging research. To ensure the quality and appropriateness of the dataset, we performed a data curation step in which we eliminated images that were excessively blurred, obscured by mucus, or contained an excessive number of air bubbles. The overall workflow of our study is depicted in Fig. 1. The first step involved preprocessing the two types of endoscopic images. During this phase, we conducted the following operations:

Image cropping and zooming: we systematically cut and zoomed the images to remove any unnecessary content and standardize their dimensions. This preparation was essential for ensuring uniformity in the data that would be fed into our subsequent analytical models.

Patient information removal: we applied a process to anonymize the images by removing any patient-specific information, thereby complying with privacy and ethical considerations.

Border trimming: unnecessary black borders surrounding the images were removed to enhance the quality and consistency of the dataset.

After this preprocessing, we harnessed hyperspectral imaging technology to transform the white light endoscopic images into spectral data. The conversion process was described in detail in "Hyperspectral imaging algorithm" of the paper.

In essence, this transformation involved mapping the visible light spectrum (ranging from 380 to 780 nm) into 401 distinct spectral bands. To do this, we employed a set of 24 color reference blocks as targets for calibration. Both a spectrometer and the endoscope were employed to capture the spectral information and images of these reference blocks. The collected data, comprising spectral information and images from these 24 color blocks, was then subjected to further analysis. A key aspect of this analysis involved the derivation of transformation matrices for each color space utilized in the study. These transformation matrices allowed us to convert RGB images into spectral information, a crucial step in our research. This detailed process of data preprocessing, spectral conversion, and transformation matrix generation was central to our study and formed the foundation for subsequent analysis and model development.

### Data preprocessing

WLI images were acquired using the Olympus Evis Lucera CV-260 SL endoscopy system, and NBI images were captured using the Olympus Evis Lucera CLV-260 endoscopy system. These two distinct endoscopy systems were selected to ensure comprehensive image acquisition and analysis in our study. The Olympus Evis Lucera CV-260 SL, equipped with its specific imaging technology, provided high-quality conventional white light images, while the Olympus Evis Lucera CLV-260, designed for narrow-band imaging, offered specialized images that highlight specific tissue characteristics through the use of narrow spectral bands. This deliberate selection of endoscopy systems allowed us to capture a diverse range of images, catering to the objectives of our research and enabling a more detailed and accurate analysis of esophageal cancer cases. Data cleansing was performed on the images in the database, and then the image was trimmed to remove unnecessary noise, black borders, and patient information, leaving an image of the esophagus, which was then uniformly scaled to a 380 × 380-pixel image size.

### Hyperspectral imaging algorithm

In this paper, the visible light band (380–780 nm) was used to simulate the preprocessed WLI images into NBI images using HSI conversion technology. The HNBI technique used in this study was developed by Hitspectra Intelligent Technology Co., Ltd, Taiwan based on the spectrum aided visual enhancer (SAVE). More detailed description of hyperspectral imaging algorithm was shown as S5 of supplementary information. To have the same standard between the spectrometer and the endoscope, the standard 24 color blocks were used as the conversion shooting benchmark. To obtain the conversion matrix between the endoscope (OLYMPUS EVIS LUCERA CV-260 SL) and the spectrometer (Ocean Optics, QE65000), both instruments captured the image to obtain its chromaticity value, and the spectrum was measured to obtain its spectrum value. Through data processing the conversion matrix was derived. The overall modeling architecture flow chart is shown in Fig. 1. In the endoscope part, sRGB color gamut space is converted to the XYZ color gamut space using Eq. (1).

1 $$\left[\begin{array}{c}X\\ Y\\ Z\end{array}\right]=\left[{M}_{A}\right]\left[T\right]\left[\begin{array}{c}f\left({R}_{sRGB}\right)\\ f\left({G}_{sRGB}\right)\\ f\left({B}_{sRGB}\right)\end{array}\right]\times 100 , 0\le \genfrac{}{}{0pt}{}{{R}_{sRGB}}{\begin{array}{c}{G}_{sRGB}\\ {B}_{sRGB}\end{array}} \le 1$$

Because the endoscope image stores data in accordance with the sRGB color gamut space standard format, the red, green, and blue (RGB) values (0–255) of the endoscope image needed to be converted to a small-scale range (0–1). The gamma function converts the sRGB value into a linear RGB value and when combined with the conversion matrix (T), the linear RGB value can be converted to the XYZ values normalized by the gamut space. However, in the conversion, the chromatic adaptation transformation matrix must be used to make corrections because the standard white point of the sRGB color gamut space is D65 (*XCW, YCW,* and *ZCW*), which is different from the white point (*XSW, YSW,* and *ZSW*) of the measured light source. Thus, the real XYZ value (*XYZ*_*Endoscope*_) under the measured light source can be obtained through the chromatic adaptation transformation matrix. In the spectrometer part, the light source spectrum *(S(λ))* and the XYZ color matching function *(x ®(λ)、y ®(λ)、z ®(λ))* need to be combined. The *Y* value of the XYZ color gamut space is proportional to the brightness, and the upper limit of the *Y* value is 100. Using the Y value in Eq. (2), the maximum brightness and the overall brightness ratio (*k*) of the light source spectrum can be calculated. The reflection spectrum data can be obtained from Eq. (3).

2 $$k=100/{\int }_{380\, nm}^{780\, nm}S\left(\lambda \right)\overline{y }\left(\lambda \right)d\lambda$$

$$X=k{\int }_{380\, nm}^{780\, nm}S\left(\lambda \right)R\left(\lambda \right)\overline{x }\left(\lambda \right)d\lambda$$

3 $$Y=k{\int }_{380\, nm}^{780\, nm}S\left(\lambda \right)R\left(\lambda \right)\overline{y }\left(\lambda \right)d\lambda$$

$$Z=k{\int }_{380\, nm}^{780\, nm}S\left(\lambda \right)R\left(\lambda \right)\overline{z }\left(\lambda \right)d\lambda$$

Many errors could contribute to this process such as nonlinear response, dark current, inaccurate color separation of color filters, and color shift. Therefore, a correction coefficient matrix (*C*) was obtained by carrying out multiple regression analysis using Eq. (4). The correction matrix was multiplied with a variable V matrix to obtain the corrected X, Y, and Z values (*XYZ*_*Correct*_).

4 $$\left[\mathrm{C}\right]=\left[{\mathrm{XYZ}}_{\rm{Spectrum}}\right]\times \mathrm{pinv}(\left[\mathrm{V}\right])$$

The root-mean-square error (RMSE) of the data of *XYZ*_*Correct*_ and *XYZ*_*Spectrum*_ were calculated, and the average errors of WLI and NBI endoscopes are 1.40 and 2.39, respectively. Using the XYZ values of the 24 color patches obtained from *XYZ*_*Correct*_ and the reflection spectrum data of the 24 color patches measured by the spectrometer (*R*_*Spectrum*_), the transformation matrix (*M*) was computed. Then, principal component analysis was performed on *R*_*Spectrum*_ of the 24 color blocks to find the main components and compare the corresponding principal component scores with the *XYZ*_*Correct*_ to obtain the conversion matrix. Finally, by comparing the obtained 24-color patch simulation spectrum (*S*_*Spectrum*_) with the *R*_*Spectrum*_, the RMSE and error of each color patch were determined. The average error of WLI is 0.057, and the average error of NBI is 0.097. The difference between *S*_*Spectrum*_ and the *R*_*Spectrum*_ can be expressed by color difference. The average color differences between WLI and NBI are 2.85 and 2.60.

The visible light hyperspectral process and technology can calculate and simulate the reflection spectrum from the RGB values captured by the monocular camera. If the RGB values of the entire image are calculated through hyperspectral technology, an HSI can be obtained.

### Band selection

**Figure 2:**
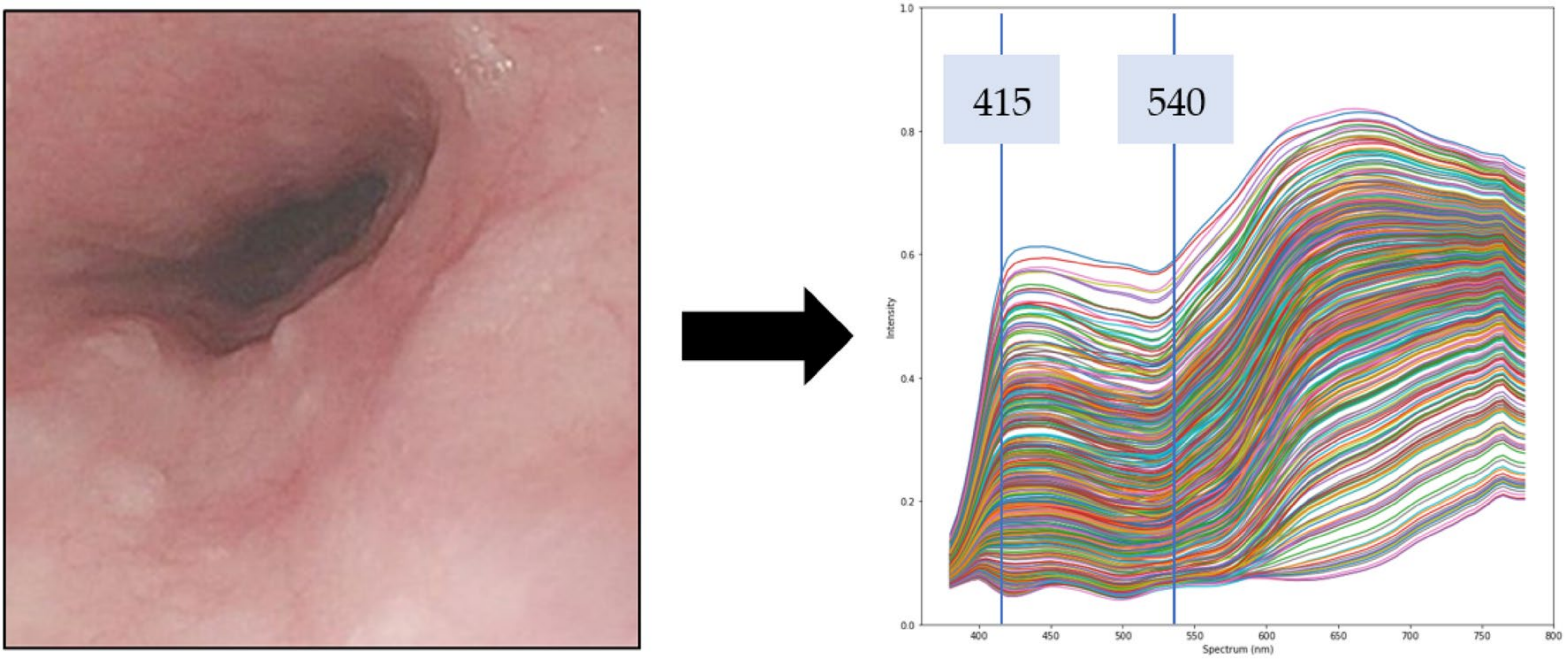
Schematic diagram of band selection.

Using hyperspectral techniques, HSI image within bandwidth of 380–780 nm (401 bands) was generated. In order to simulate NBI images, this study uses the band of 415 nm and 540 nm. 415 nm wavelength can allow the light to be absorbed by heme, and with low penetrating power for the superficial mucosal tissue, which is advantageous for blood vessel observation of superficial mucosal. The microvessels will appear brown. Using the 540 nm band is conducive to distinguishing superficial mucosal tissue lesions. The blood vessels in the submucosal tissue are blue. The use of the above two bands can create a strong sense of hierarchy, which is more conducive to identifying mucosal tissue lesions. Figure 2 is a schematic diagram of band selection.

### CycleGAN model parameter settings

In this paper, CycleGAN was used to generate the CNBI image, and Pytorch was used as its DL framework. Adversarial loss, cycle consistency loss, and equilibrium were used as the loss functions. The sum of the loss known as the identity loss is shown in Eq. (5). The batch size was setx to 1, and the initial learning rate was 0.0002, which decreases as the number of training times increases. More detailed description of CycleGan was shown as S1 of supplementary information.

5 $$L\left(G, F,{D}_{X},{D}_{Y}\right)={L}_{GAN}\left(G,{D}_{Y},X,Y\right)+{L}_{GAN}\left(F,{D}_{x},Y,X\right)+{\lambda }_{cycle}{ L}_{consitency}\left(G,F\right)+{\lambda }_{id}{ L}_{identity}\left(G,F\right)$$

### Metrics used for evaluation

CIEDE2000 is a color difference formula that quantifies the perceptual difference between two colors. It is based on the CIELAB color space. The equation for CIEDE2000 is shown in Eq. (6). More detailed description of CIEDE2000 was shown as S2 of supplementary information.

6 $$CIEDE 2000= \sqrt{{{\left(\frac{\mathrm{\Delta L{\prime}}}{kL}\right)}^{2}+{\left(\frac{\mathrm{\Delta C{\prime}}}{kC}\right)}^{2}+{\left(\frac{\mathrm{\Delta H{\prime}}}{kH}\right)}^{2}+R\frac{\mathrm{\Delta C{\prime}}}{kC}\frac{\mathrm{\Delta H{\prime}}}{kH} }^{.}}$$

Here, *ΔL', ΔC', and ΔH'* represent differences in lightness, chroma, and hue, respectively, between two colors. The constants *kL, kC, and kH* are scaling factors, and *R* is a rotational term. The range of CIEDE2000 typically falls between 0 and 100, with lower values indicating smaller color differences, and higher values indicating larger differences. Entropy is a measure of the amount of information or randomness in an image. The equation for entropy is shown in Eq. (7). More Entropy description of CycleGan was shown as S3 of supplementary information.

7 $$\sum p\left(x\right).{\mathrm{log}}_{2}p(x)$$

Here, *H(X)* represents the entropy of the image, and *p(x)* is the probability of occurrence for each pixel value x. Entropy values range from 0 to the maximum value of the information source (usually 8 bits, i.e., 0 to 255 for images). Higher entropy values indicate greater information content or randomness. SSIM quantifies the structural similarity between two images. The equation for SSIM is shown in Eq. (8).

8 $$SSIM= \frac{\left(2\mathrm{\mu x\mu y}+\mathrm{C}1\right)(2*\mathrm{\sigma xy }+\mathrm{ C}2) }{({\mathrm{\mu x}}^{2} + {\mathrm{\mu y }}^{2}+\mathrm{ C}1) * ({\mathrm{\sigma x }}^{2}+ {\mathrm{\sigma y }}^{2}+\mathrm{ C}2)}$$

Here, *μx* and *μy* represent the means of the two images, *σx* and *σy* are the standard deviations, and *σxy* is the covariance. *C1* and C2 are small constants added for numerical stability. SSIM values range from -1 to 1, where 1 indicates perfect similarity, 0 suggests no similarity, and -1 denotes perfect dissimilarity. More detailed description of SSIM was shown as S4 of supplementary information.

### Institutional review board statement

In this study, all image data provided by the Kaohsiung Armed Forces General Hospital are completely removed from personal data information. A statement confirming that informed consent is not required to have been obtained from all subjects and/or their legal guardians, according to the guidelines of the Declaration of Helsinki, and approved by the Institutional Review Board of Kaohsiung Armed Forces General Hospital (KAFGHIRB 112-018).

## Results and discussions

**Figure 3:**
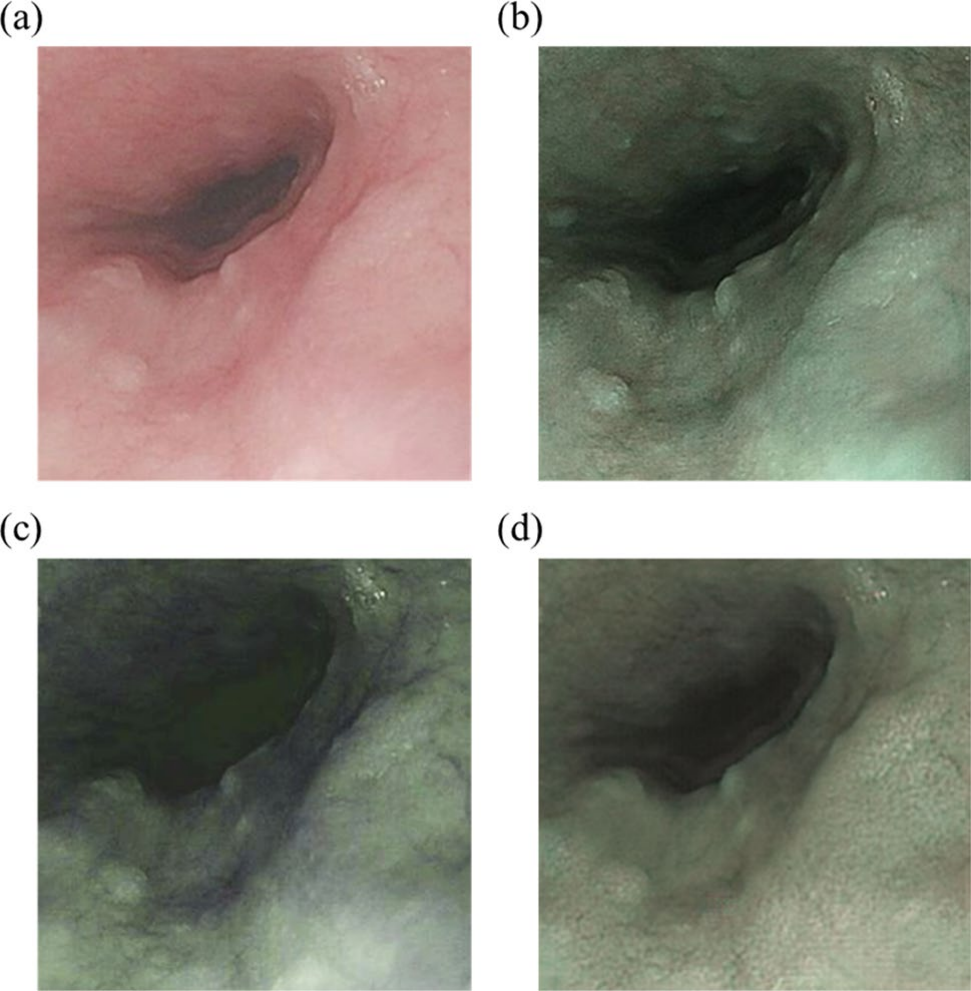
Images of WLI (**a**), NBI (**b**), HNBI (**c**), and CNBI (**d**).

This section uses WLI, NBI, HNBI, and CNBI, as shown in Fig. 3, to evaluate and compare the objective indicators of CIEDE2000, entropy, and SSIM and interpret the results. We chose these metrics due to their relevance to the primary research objective, which is the detection of early-stage esophageal cancer using various imaging techniques. SSIM assesses structural similarities between images, which is essential for detecting anomalies in medical images. Entropy effectively measures information content in images, while CIEDE2000 is well-suited for evaluating color differences. Firstly, these metrics are widely recognized and accepted within the field of image analysis and medical imaging, ensuring the compatibility of the findings with existing research^52^. Secondly, these metrics are particularly suited to the assessment of image quality and characteristics relevant to our study, such as structural similarities, information content, and color differences. Moreover, the practicality and efficiency of these metrics were crucial considerations, as they allowed for a focused and meaningful evaluation without compromising the depth of our analysis.

### CIEDE2000 result evaluation

Table 1 shows the results of the color difference between the blood vessels and the esophagus background tissue of each of the four images of WLI, NBI, CNBI, and HNBI. The total number of images tested is 100, and the size of each image is adjusted to 380 × 380. The minimum, average, and maximum images are listed. The WLI color difference values are 2.07, 8.24 and 14.91. The NBI color difference values are 5.85, 12.29 and 20.71. The CNBI color difference values are 5.91, 12.86 and 20.13. The HNBI color difference values are 6.43, 13.61, and 21.06.

**Table 1:** Color difference comparison table of WLI, NBI, CNBI, and HNBI.

Images	CIEDE 2000 Minimum	CIEDE 20000 Average	CIEDE 2000 Maximum
WLI	2.07	8.24	14.91
NBI	5.85	12.29	20.71
CNBI	5.91	12.86	20.13
HNBI	6.43	13.61	21.06

### Entropy result evaluation

Table 2 shows the entropy comparison results of the four images of WLI, NBI, CNBI, and HNBI. The minimum, average, and maximum values are listed. The entropy values of WLI are 5.31, 6.47, and 7.37. The order of entropy values of NBI is 5.11, 6.15, and 7.19. The order of entropy values of CNBI is 4.71, 5.67, and 6.62. The order of entropy values of HNBI is 3.39, 4.77, and 6.18.

**Table 2:** Entropy comparison table of WLI, NBI, CNBI, and HNBI.

Images	Entropy (bits) Minimum	Entropy (bits) Average	Entropy (bits) Maximum
WLI	5.31	6.47	7.37
NBI	5.11	6.15	7.19
CNBI	4.71	5.67	6.62
HNBI	3.39	4.77	6.18

### SSIM result evaluation

Table 3 shows the comparison results of SSIM similarity between WLI, NBI, HNBI, and CNBI. The number images used for evaluations is 74. The minimum, average, and maximum values of SSIM are presented. The SSIM values of NBI compared with HNBI are 0.61, 0.68, and 0.8. The SSIM values of NBI and CNBI are 0.57, 0.64, and 0.76.

**Table 3:** Comparison table of SSIM similarity between NBI, CNBI, and HNBI.

Images	SSIM Minimum	SSIM Average	SSIM Maximum
WLI	0.61	0.68	0.8
NBI	0.57	0.64	0.76

**Figure 4:**
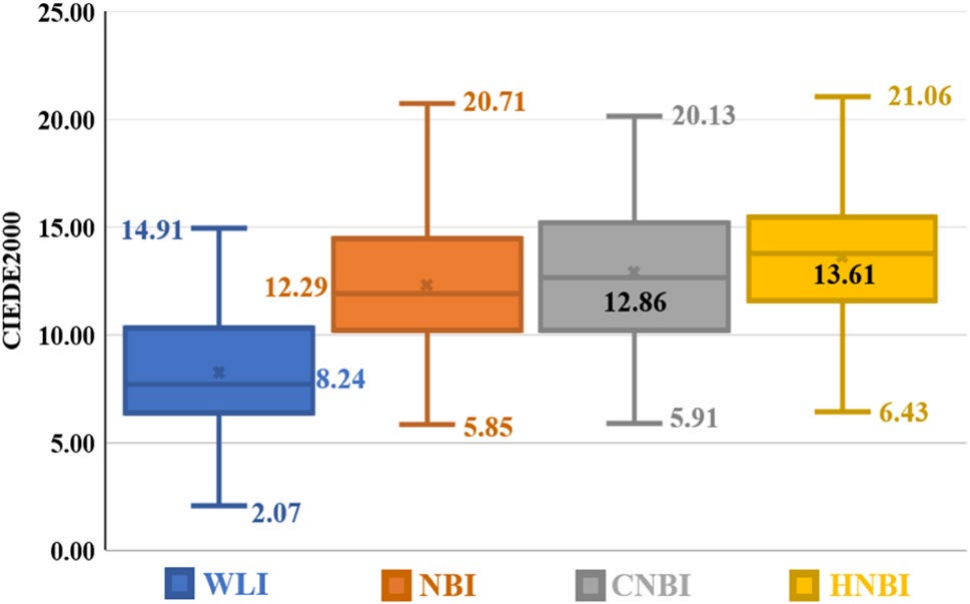
Statistical chart of color difference comparison results.

**Figure 5:**
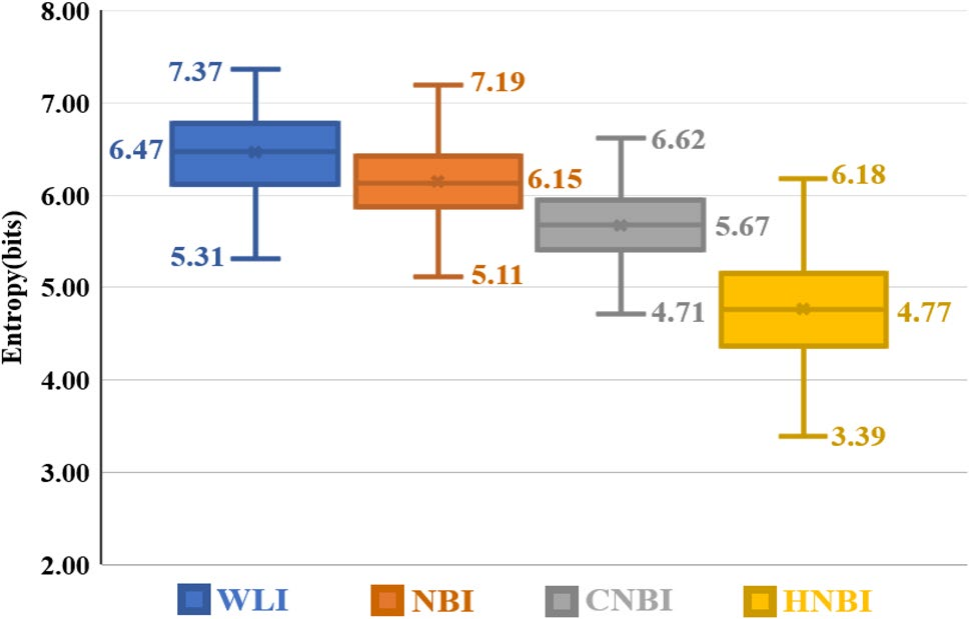
Statistical graph of entropy comparison results.

**Figure 6:**
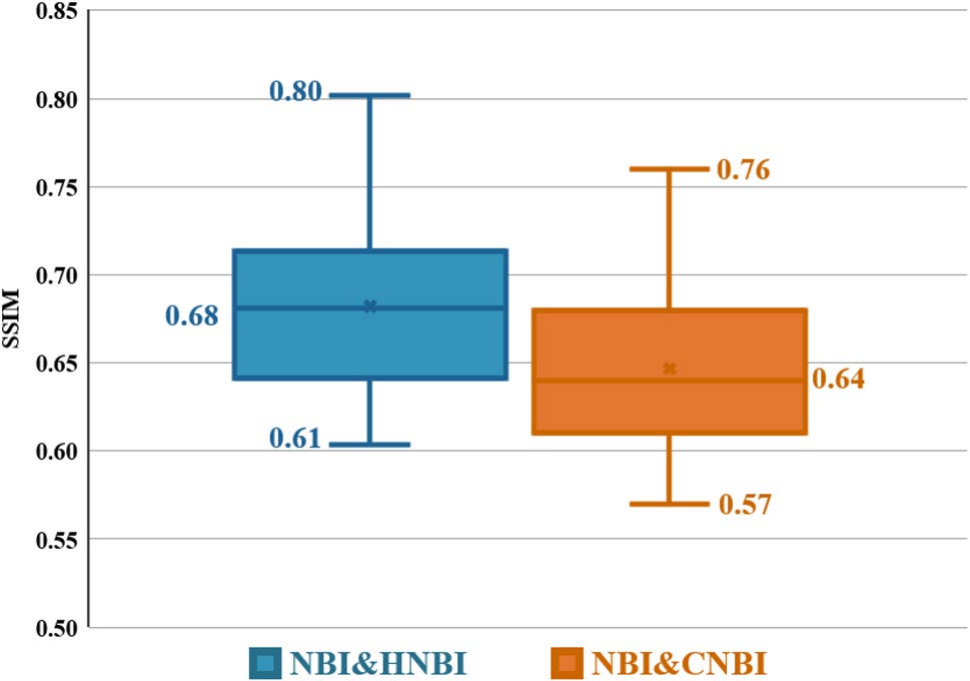
Statistical chart of SSIM similarity comparison results between NBI, CNBI, and HNBI.

Figure 4 is a statistical chart of the color difference comparison results between the blood vessels and the esophagus background tissue of each of the four images of WLI, NBI, CNBI, and HNBI. The tissue chromatic aberration is low in WLI. The esophageal background tissue color difference of HNBI blood vessels is slightly higher than that of NBI and CNBI. Figure 5 is a statistical chart of the entropy comparison results of the four images of WLI, NBI, CNBI, and HNBI. The entropy value of HNBI is generally lower than that of WLI, NBI, and CNBI. Figure 6 is a statistical chart of the comparison results of SSIM between WLI, NBI, CNBI, and HNBI. The similarity between HNBI and NBI is slightly higher than that of CNBI and NBI, which shows HNBI has more advantages in simulating NBI.

The combination of HSI with NBI via band selection enables the creation of HNBI pictures, which exhibit improved capability in differentiating blood vessels within these images. Moreover, the use of CNBI technology proves to be a vital asset in the realm of AI for enhancing object recognition in images. This technique effectively minimizes the duration needed for picture acquisition and preprocessing. This discovery has the potential to be effectively incorporated into contemporary endoscopic cameras, such as tiny capsule endoscopes that do not include narrowband filters. Consequently, it may enhance patient comfort and optimize the efficiency of endoscopic treatments. In addition, the use of a 256 × 256 picture resolution for emulating narrowband images, accompanied by efficient conversion rates, guarantees compatibility with diverse hardware capabilities, hence facilitating the integration of this approach into a wide array of endoscope systems. The aforementioned developments possess the capacity to augment diagnostic precision for healthcare professionals and expedite the identification of early symptoms, hence diminishing the likelihood of patient fatality. Notwithstanding these benefits, it is essential to address certain constraints of our suggested approach. The efficacy of our methodology may be subject to the impact of hardware capabilities, leading to variances in conversion performance across diverse configurations. Furthermore, it should be noted that the potential effectiveness of HNBI pictures is contingent upon the particular demands of the diagnostic undertaking and the apparatus used. Moreover, while CNBI technology demonstrates effective image augmentation, its applicability to various datasets and clinical contexts need additional research. Like any other technology advancement, the effective incorporation of these techniques into clinical settings may need continuous refinement and verification, such as conducting rigorous clinical studies and evaluating their usefulness.

## Conclusions

The purpose of this paper is to verify the effectiveness of NBI images simulated by the combination of HSI and band selection by using four different images: WLI, NBI, CNBI, and HNBI. The effectiveness of distinguishing blood vessels in this type of images are studied by three indicators: entropy, color difference, and SSIM. HSI combined with NBI has relatively satisfactory performance. The NBI images simulated with CycleGAN can be used in AI object detection image augmentation to reduce the time required for image collection and preprocessing. This simulated NBI technology can be used along with the newer endoscopic cameras such as capsule endoscopic cameras that cannot be fitted with narrowband filters, thereby reducing the discomfort for the patients and the time required for endoscopy. At the same time, new endoscopes, such as capsule endoscopes, in order to improve the convenience of entering the body and reduce the discomfort of patients, are designed to be in the size of capsules, which are not suitable for carrying NBI filters. The 256 × 256 image size is used to simulate narrow-band images, and it is found that the conversion speed of CycleGAN is about 3 fps, and the hyperspectral conversion speed is about 25 fps. With different hardware facilities, there will be different conversion performance. Therefore, the hyperspectral simulated narrow-band images in this study can be matched with new endoscopes. It is believed that it can provide doctors with better recognition rates in the future. If the technology of this study continues to develop, it can also be used with artificial intelligence object detection, not only provide patients with better comfort, but may also be able to detect symptoms earlier and reduce the risk of patient death.

### Supplementary Information

Supplementary Information.

## Data Availability

The datasets used and/or analyzed during the current study available from the corresponding author on reasonable request.
