# Supplementary material for: Assessment of hyperspectral imaging and CycleGAN-simulated narrowband techniques to detect early esophageal cancer
Source: Sci Rep. 2023 Nov 22;13:20502. doi: 10.1038/s41598-023-47833-y (PMC10665456; doi:10.1038/s41598-023-47833-y)
Supplement: Supplementary file 1 — Supplementary Information. [file 41598_2023_47833_MOESM1_ESM.docx]

**Assessment of Hyperspectral Imaging and CycleGAN-Simulated Narrowband Techniques to Detect Early Esophageal Cancer: Supplementary Material**

Kai-Yao Yang ^1^, Arvind Mukundan ^2^, Yu-Ming Tsao ^2^, Xian-Hong Shi ^2^, Chien-Wei Huang ^1,3,*^ , and Hsiang-Chen Wang ^2,4,5,^*

1 Department of Medical Material Research, Kaohsiung Armed Forces General Hospital, 2, Zhongzheng 1st.Rd., Lingya District, Kaohsiung City 80284, Taiwan. yangkaiyao@gmail.com (K.Y.Y.); forevershiningfy@yahoo.com.tw (C.-W.H.)

2 Department of Mechanical Engineering, National Chung Cheng University, 168, University Rd., Min Hsiung, Chia Yi County 62102, Taiwan. d09420003@ccu.edu.tw (A.-M.); d09420002@ccu.edu.tw (Y.-M.T.); a0983906104@gmail.com (X.-H.S.)

3 Department of Nursing, Tajen University, 20, Weixin Rd., Yanpu Township, Pingtung County 90741, Taiwan.

4 Director of Technology Development, Hitspectra Intelligent Technology Co., Ltd., 4F., No. 2, Fuxing 4th Rd., Qianzhen Dist., Kaohsiung City 80661, Taiwan.

5 Department of Medical Research, Dalin Tzu Chi General Hospital, 2, Min-Sheng Rd., Dalin Town, ChiaYi 62247, Taiwan

* Correspondence: Chien-Wei Huang (forevershiningfy@yahoo.com.tw) ; Hsiang-Chen Wang ([hcwang@ccu.edu.tw](mailto:hcwang@ccu.edu.tw))

**Abstract**

This article provides an overview of the supplementary material for the article Evaluating Hyperspectral Techniques Using Objective Metric Research on Analog Narrowband Image.

*Keywords*: esophageal cancer, hyperspectral imaging, band selection, color reproduction, CycleGAN, CIEDE2000, entropy, SSIM

**1. CycleGAN**

Image conversion technology generally requires a large amount of paired data. However, collecting paired data is often time consuming and labor intensive. In 2017, Jun-Yan Zhu et al. proposed Cycle-Consistent Adversarial Networks (CycleGAN). [1]. CycleGAN consists of generator architecture and discriminator architecture. This algorithm does not need paired data, it can automatically perform image-to-image conversion technology, and it can be blurred, color converted, improve clarity, or fill in missing blanks. During training, the loss function is adversarial loss, cycle consistency loss, and the sum of identity loss, and then trained according to its size.

**2. CIEDE 2000**

CIE Technical Committee TC 1-47 Hue and brightness related correction for industrial color difference evaluation was established in October 1998 to improve the performance of CIE94 color difference formula. In 2001, CIE redefined the color difference calculation method. CIEDE2000 is the latest color difference formula [2]. This formula is much more complicated than CIE94, but it greatly improves the accuracy, so that in the entire CIELAB color space, the original ellipse becomes more like a circle.

Because the use of CIEDE2000 requires the Lab value of the Lab color space in the image, the previously preprocessed white light image (WLI) and narrowband imaging (NBI) images and the NBI simulated by band selection through the mask is used to obtain the average R, G, and B values of WLI, NBI, simulated NBI vessels, and esophageal background tissue; convert the obtained R, G, and B values to the XYZ color gamut space through Equation S1; and then convert to the Lab color gamut space through Equation S2 to obtain the average Lab value of WLI, NBI, and simulated narrowband image blood vessel images and the image average Lab value of esophageal background tissue.

After obtaining the Lab values of each image, the CIEDE2000 Equation (S3) can be used to obtain the contrast color difference between the blood vessels and the esophageal background tissue in a single image and further compare the contrast differences between images, where KL, KC, and KH are the coefficient weights; RT is the hue rotation item used to correct the blue area of the color space; SL, SC, and SH are the brightness compensation, chroma compensation, and hue compensation, respectively.

| $\left\lceil\begin{aligned} X \\ Y \\ Z \end{aligned} \right\rceil=\left[ \begin{matrix} 0.4124564 & 0.3575761 & 0.1804375 \\ 0.2126729 & 0.7151522 & 0.0721750 \\ 0.0193339 & 0.1191920 & 0.9503041 \end{matrix} \right]\left[ \begin{aligned} R \\ G \\ B \end{aligned} \right]$ | （S1） |
| --- | --- |
| $\Delta E_{00}=\sqrt{\left( \frac{\Delta L^{'}}{K_{L}S_{L}} \right)^{2}+\left( \frac{\Delta{C_{ab}}^{'}}{K_{c}S_{c}} \right)^{2}+\left( \frac{\Delta{H_{ab}}^{'}}{K_{H}S_{H}} \right)^{2}+R_{T}\left( \frac{\Delta{C_{ab}}^{'}}{K_{c}S_{c}} \right)\left( \frac{\Delta{H_{ab}}^{'}}{K_{H}S_{H}} \right)}$ | （S2） |
| $\Delta L^{'}={L^{'}}_{1}-{L^{'}}_{2}$ |  |
| $\Delta{C_{ab}}^{'}={C^{'}}_{2}-{C^{'}}_{1}$ | （S3） |
| $\Delta{H_{ab}}^{'}=2\sqrt{{{{C^{'}}_{1}C}^{'}}_{2}}\sin\left( \frac{\Delta{h^{'}}_{ab}}{2} \right)$ |  |

**3. Entropy**

For images, the theoretical basis of information entropy is used. Because the grayscale of each pixel in the image can change naturally, the definition of information entropy is extended to the image field, and the number of times a certain grayscale level appears in the entire image is compared with the number of the entire image. For an 8-bit image, the grayscale range is 0–255 for a total of 256. First, the statistics of the grayscale histogram of the image is calculated, then the probability of each grayscale level is determined, and the grayscale is further distributed. Finally, the aggregation feature of the gray distribution is used to perform the entropy operation with Equation S4.

| $H=-\sum_{i=0}^{255} \log_{2} Pi$ | (S4） |
| --- | --- |

**4. Structural similarity index evaluation**

The structural similarity index algorithm is used to compare the brightness, contrast, and structure of the narrowband image and the simulated narrowband image. In terms of brightness, the average measurement of all image pixel values is shown in Equation S5, where xi is the image, the i-th pixel value of x, and N is the total number of pixel values; in terms of contrast, it is measured by taking the standard deviation (square root of variance) of all pixel values, such as Equation S6; in terms of structure, the signal divided by its standard deviation is normalized so that the two signals being compared have unit standard deviation as in Equation S7, as a measure of structure. Finally, these three elements are weighted and expressed by the product, and the two image signals x and y are given, which are defined as Equation S8.

| $\mu_{x}=\frac{1}{N}\sum_{i=1}^{N} x_{i}$ | （S5） |
| --- | --- |

| $\sigma_{x}=\left( \frac{1}{N-1}\sum_{i=1}^{N} \left( x_{i}-\mu_{x} \right)^{2} \right)^{\frac{1}{2}}$ | （S6） |
| --- | --- |

| $\sigma_{xy}=\frac{1}{N-1}\sum_{i=1}^{N} \left( x_{i}-\mu_{x} \right)\left( y_{i}-\mu_{y} \right)$ | （S7） |
| --- | --- |

| $SSIM(x,y)=\left[ l\left( x,y \right) \right]^{\alpha}\cdot\left[ c\left( x,y \right) \right]^{\beta}\cdot\left[ s\left( x,y \right) \right]^{\gamma}$ | （S8） |
| --- | --- |

| $l\left( x,y \right)=\frac{2\mu_{x}\mu_{y}+C_{1}}{{\mu^{2}}_{x}+{\mu^{2}}_{y}+C1}$ |  |
| --- | --- |

| $c\left( x,y \right)=\frac{2\sigma_{x}\sigma_{y}+C_{2}}{{\sigma^{2}}_{x}+{\sigma^{2}}_{y}+C_{2}}$ | （S9） |
| --- | --- |

| $s\left( x,y \right)=\frac{\sigma_{xy}+C_{3}}{\sigma_{x}\sigma_{y}+C_{3}}$ |  |
| --- | --- |

However, its algorithm has the characteristics of symmetry, limitation, and uniqueness of the maximum value in use. Symmetry means the structural similarity index is symmetrical, the limitation is that its maximum value is 1, and the single maximum value also means when the two signals to be measured are exactly the same, the value of the structural similarity index is 1, and its characteristics are shown in Equations S10, S11, and S12.

| $s\left( x,y \right)=s\left( x,y \right)$ | (S10） |
| --- | --- |
| $s\left( x,y \right)\leq1$ | （S11） |
| $s\left( x,y \right)=1$ | (S12) |

When calculating the structural similarity index between the narrowband image and the simulated narrowband image, the parameters are set as α = β = γ = 1 and C3 = C2/2 to obtain the formula. To make the result more accurate, an 11×11 local small window is opened and moved pixel by pixel on the entire image. The structural similarity of the signals in each small window is calculated, and these signals are averaged to obtain the structural similarity index between the narrowband image and the simulated narrow-band image from Equation S13.

| $SSIM\left( x,y \right)=\frac{\left( 2\mu_{x}\mu_{y}+C_{1} \right)+\left( 2\sigma_{x}\sigma_{y}+C_{2} \right)}{\left( {\mu^{2}}_{x}+{\mu^{2}}_{y}+C_{1} \right)\left( {\sigma^{2}}_{x}+{\sigma^{2}}_{y}+C_{2} \right)}$ | （S13） |
| --- | --- |

**5. Hyperspectral imaging algorithm**

The visible hyperspectral imaging (VIS-HSI) used in this study is calculated by using the images taken by an endoscope combined with the visible hyperspectral algorithm (VIS-HSA). The wavelength range is from 380 nm to 780 nm, and the spectral resolution is up to 1 nm. The detailed instrument specifications used in this study are mentioned in Table 1.

The individual conversion formulas to convert the 24-colour patch image and 24 colour patch reflectance spectrum data to XYZ colour space are as follows

On the camera side: convert sRGB color gamut space to XYZ color gamut space

$\left[ \begin{aligned} X \\ Y \\ Z \end{aligned} \right]=[M_{A}]\left[ T \right]\left[ \begin{aligned} f\left( R_{sRGB} \right) \\ f\left( G_{sRGB} \right) \\ f(B_{sRGB}) \end{aligned} \right]\times100 , 0\leq{R_{sRGB} \atop\begin{aligned} G_{sRGB} \\ B_{sRGB} \end{aligned}} \leq1$ (S14)

where

$\left[ T \right]=\left[ \begin{aligned} 0.4104 0.3576 0.1805 \\ 0.2126 0.7152 0.0722 \\ 0.0193 0.1192 0.9505 \end{aligned} \right]$ (S15)

$f\left( n \right)= \left\{ \begin{aligned} {(\frac{n+0.055}{1.055})}^{2.4}, n>0.04045 \\ \left( \frac{n}{12.92} \right), otherwise \end{aligned} \right.$ (S16)

$\left[ M_{A} \right]=\left[ \begin{aligned} \frac{X_{SW}}{X_{CW}} 0 0 \\ 0 \frac{Y_{SW}}{Y_{CW}} 0 \\ 0 0 \frac{Z_{SW}}{Z_{CW}} \end{aligned} \right]$ (S18)

On the spectrometer side: convert reflection spectral data to XYZ color gamut space

$X=k\int_{380nm}^{780nm} S\left( \lambda\right)R\left( \lambda\right)\bar{x}\left( \lambda\right)d\lambda$ (S19)

$Y=k\int_{380nm}^{780nm} S\left( \lambda\right)R\left( \lambda\right)\bar{y}\left( \lambda\right)d\lambda$ (S20)

$Z=k\int_{380nm}^{780nm} S\left( \lambda\right)R\left( \lambda\right)\bar{z}\left( \lambda\right)d\lambda$ (S22)

$k=100/\int_{380nm}^{780nm} S\left( \lambda\right)\bar{y}\left( \lambda\right)d\lambda$ (S23)

The nonlinear response of the camera can be corrected by a third-order equation, and the nonlinear response correction variable is defined as V_Non-linear_.

$V_{Non-linear}=\left[ X^{3} Y^{3} Z^{3} X^{2} Y^{2} Y^{2} X Y Z 1 \right]^{T}$ (S24)

In the dark current part of the camera, the dark current is usually a fixed value and does not change with the amount of incoming light, so a constant is given as the contribution of the dark current, and the dark current correction variable is defined as V_Dark_.

$V_{Dark}=[a]$ (S25)

Finally, VColor is used as the base, and multiplied by the nonlinear response correction of V_Non-linear_, and the result is standardized within the third order to avoid excessive correction, and finally V_Dark_ is added to obtain the variable matrix V.

$V_{Color}={[XYZ XY XZ YZ X Y Z]}^{T}$ (S26)

$V=\left[ X^{3} Y^{3} Z^{3} X^{2}Y X^{2}Z Y^{2}Z XY^{2} XZ^{2} YZ^{2} XYZ X^{2} Y^{2} Y^{2} XY XZ YZ X Y Z a \right]^{T}$ (S27)

Before using CIE DE2000 to calculate color difference, XYZ_Correct_ and XYZ_Spectrum_ must be converted from XYZ color space to lab color space. The conversion formula is as follows:

${L^{*} = 116f\left( \frac{Y}{Y_{n}} \right)-16 \atop\begin{aligned} a^{*} = 500\left[ f(\frac{X}{X_{n}})-f(\frac{Y}{Y_{n}}) \right] \\ b^{*} = 200\left[ f(\frac{Y}{Y_{n}})-f(\frac{Z}{Z_{n}}) \right] \end{aligned}}$ (S28)

$f\left( n \right)= \left\{ \begin{aligned} n^{\frac{1}{3}}, n>0.008856 \\ 7.787n+0.137931, otherwise \end{aligned} \right.$ (S29)


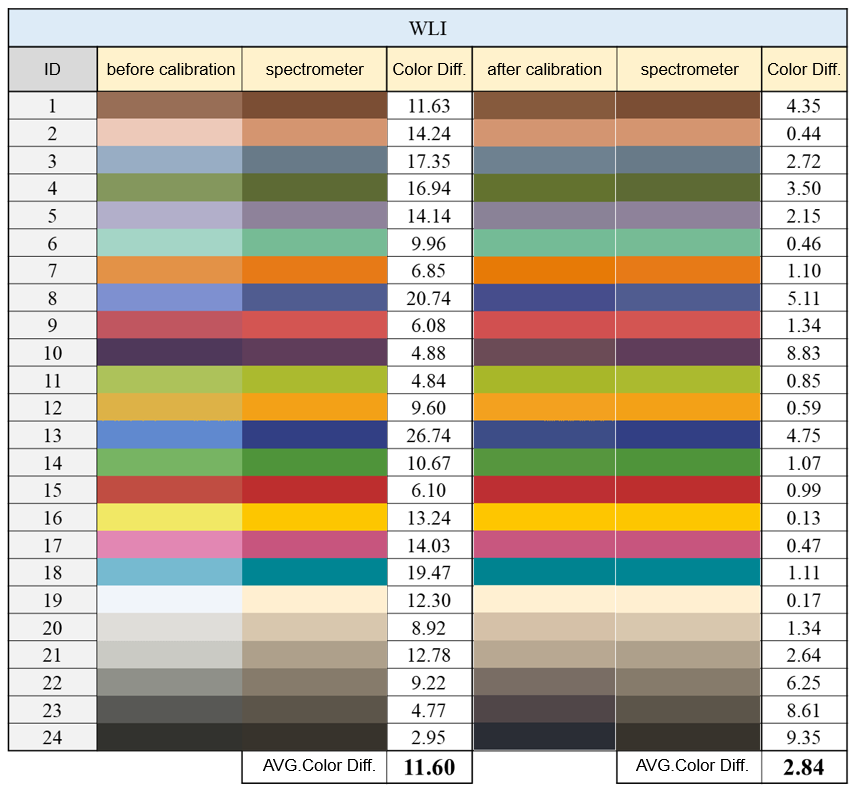


Figure S1. Comparison of chromatic aberration between WLI endoscope before and after correction and spectrometer


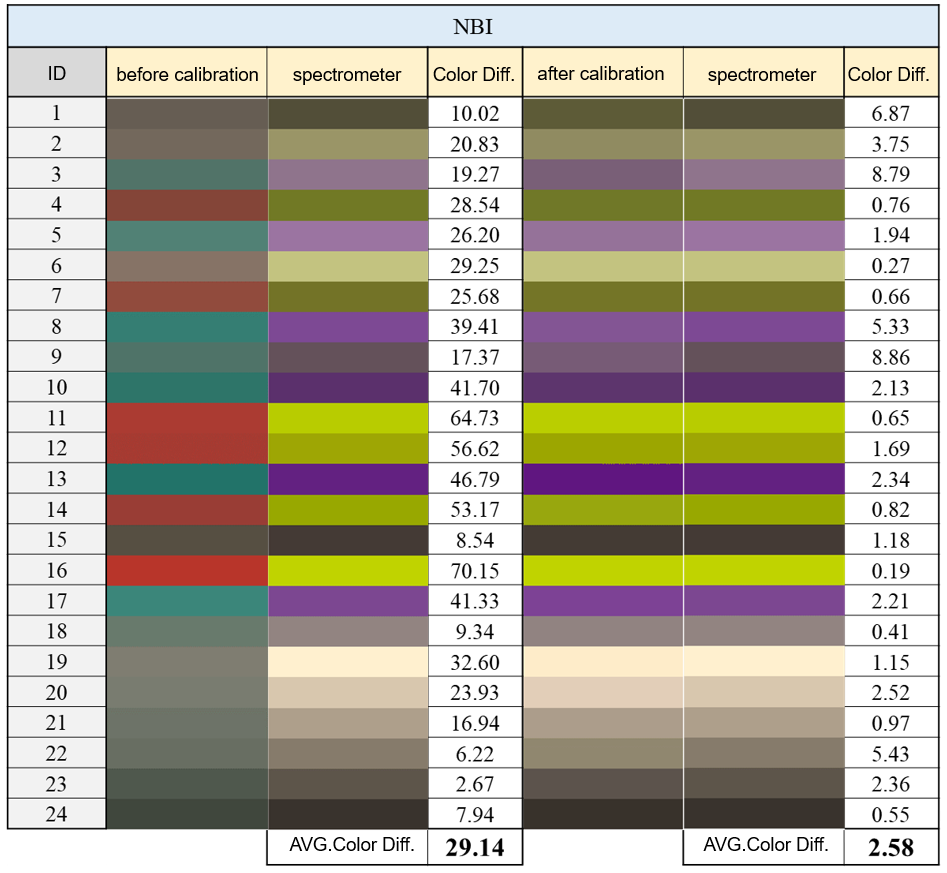


Figure S2. Comparison of chromatic aberration between NBI endoscope before and after correction and spectrometer


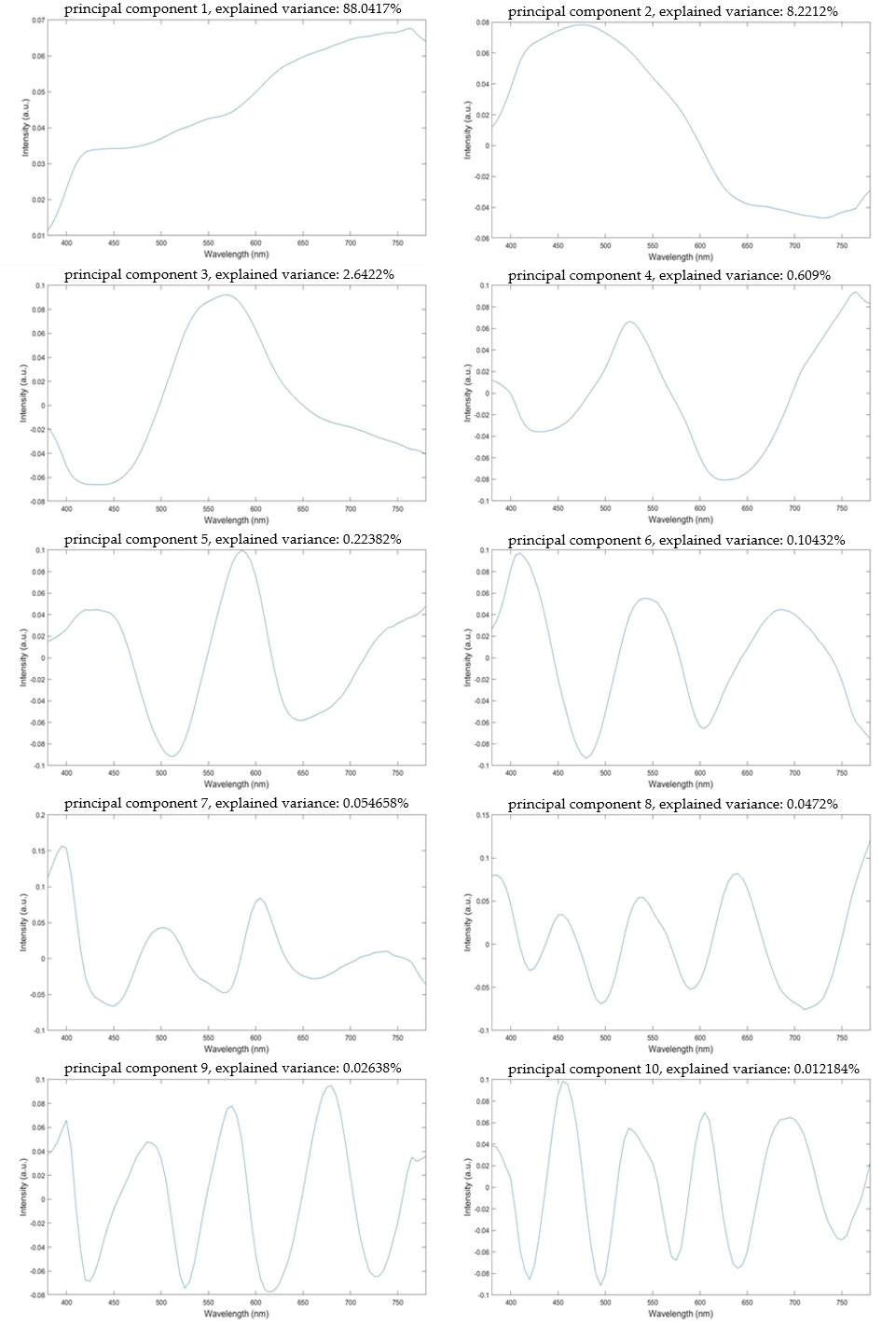


Figure S3. First 10 principal components of Rspectrum


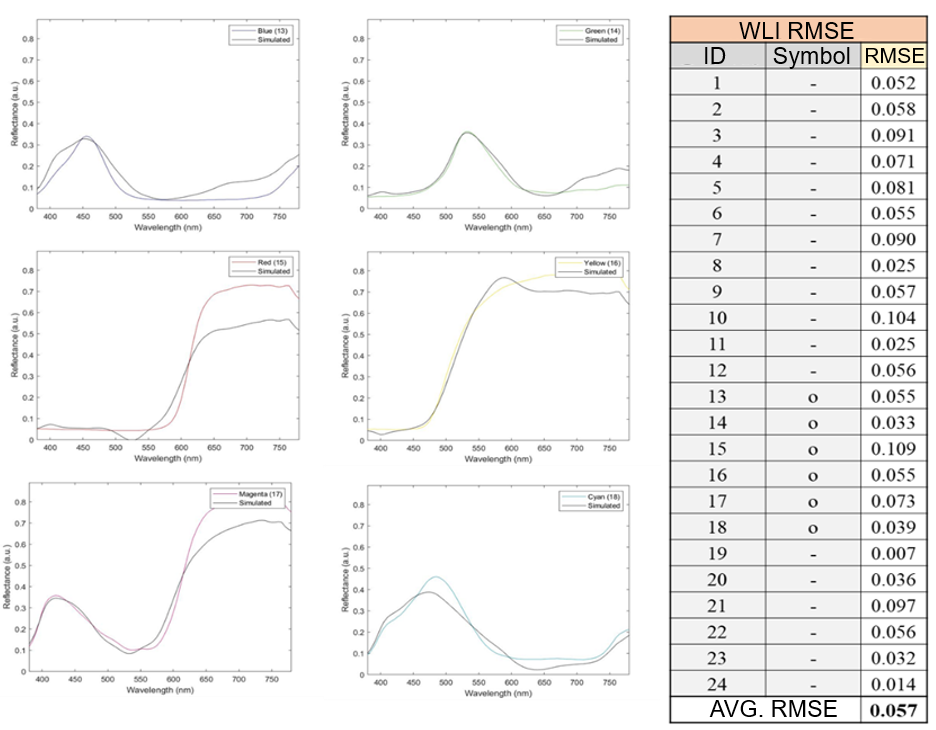


Figure S4. Root mean square error of WLI endoscope SSpectrum and Rspectrum


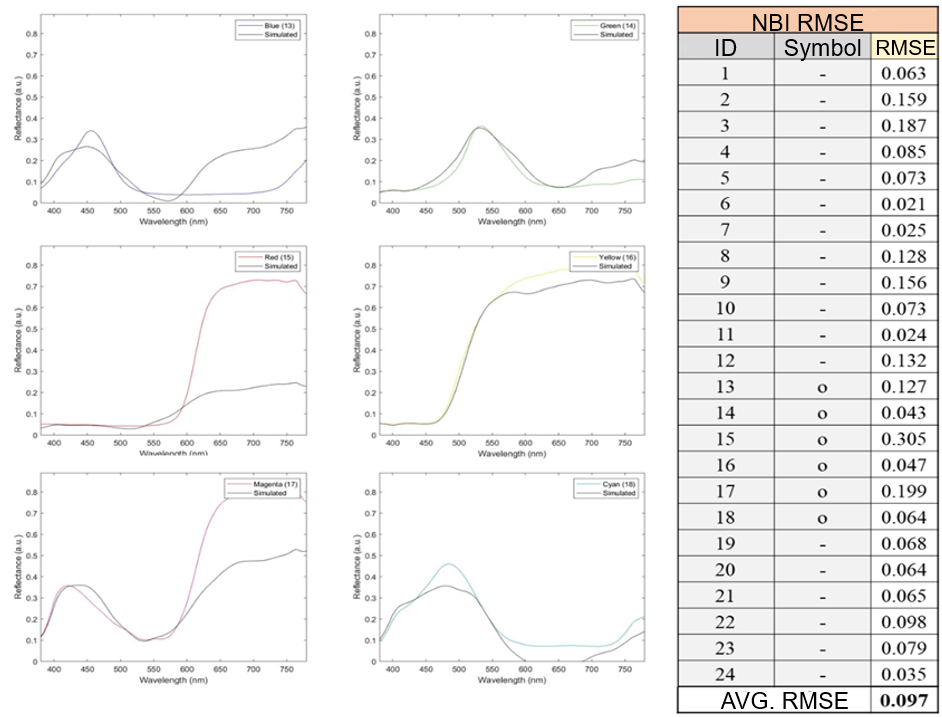


Figure S5. Root mean square error of NBI endoscope SSpectrum and RSpectrum


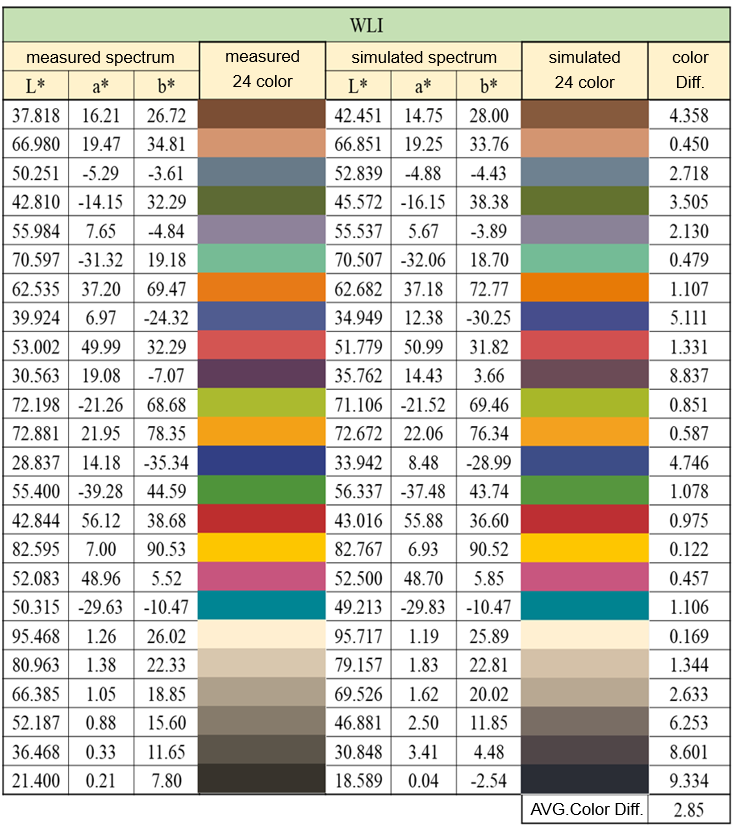


Figure S6. Chromatic difference diagram of measured spectrum and simulated spectrum of 24 color blocks of WLI endoscope


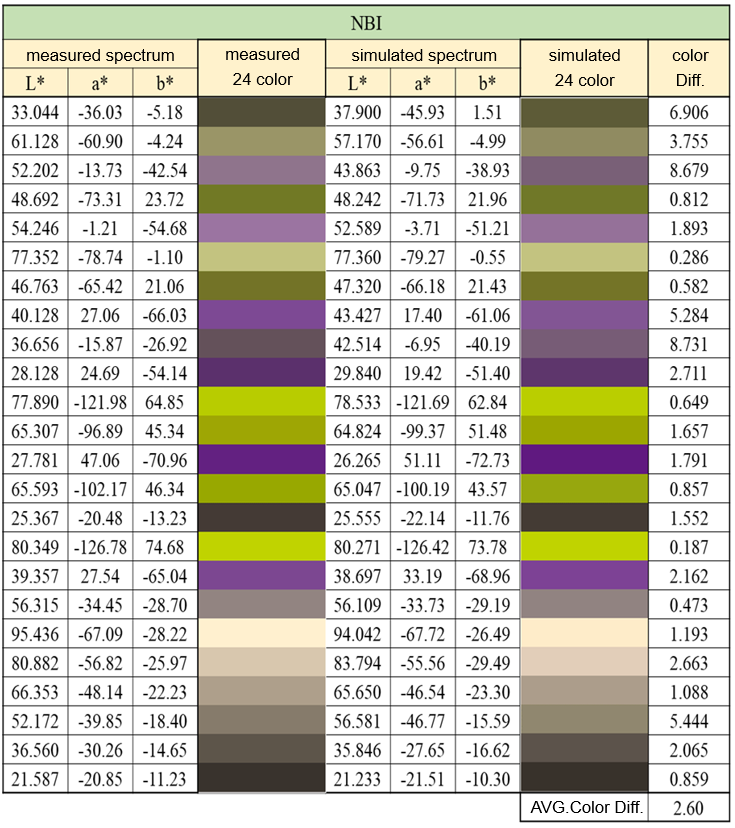


Figure S7. NBI endoscope 24-color block measurement spectrum and simulated spectrum color difference diagram
